# Rapid and Simple TLC-Densitometric Method for Assay of Clobetasol Propionate in Topical Solution †

**DOI:** 10.3390/molecules22111888

**Published:** 2017-11-03

**Authors:** Malgorzata Dolowy, Violetta Kozik, Andrzej Bak, Josef Jampilek, Krzysztof Barbusinski, Maciej Thomas, Alina Pyka-Pajak

**Affiliations:** 1 Department of Analytical Chemistry, School of Pharmacy with Division of Laboratory Medicine in Sosnowiec, Medical University of Silesia in Katowice, Jagiellonska 4, 41-200 Sosnowiec, Poland; apyka@sum.edu.pl; 2 Institute of Chemistry, University of Silesia, Szkolna 9, 40-006 Katowice, Poland; violetta.kozik@us.edu.pl (V.K.); andrzej.bak@us.edu.pl (A.B.); 3 Department of Pharmaceutical Chemistry, Faculty of Pharmacy, Comenius University, Odbojarov 10, 832 32 Bratislava, Slovakia; 4 Institute of Water and Wastewater Engineering, Silesian University of Technology, Konarskiego 18, 44-100 Gliwice, Poland; krzysztof.barbusinski@polsl.pl; 5 Chemiqua Company, Skawinska 25/1, 31-066 Krakow, Poland; biuro@chemiqua.pl

**Keywords:** clobetasol, clobetasol propionate, topical steroids, TLC-densitometry, validation

## Abstract

A rapid, simple to use and low-cost thin-layer chromatographic procedure in normal phase system with densitometric detection at 246 nm was carefully validated according to the International Conference on Harmonisation (ICH) guidelines for assay of clobetasol propionate in topical solution containing clobetasol propionate in quantity 0.50 mg/mL. The adopted thin-layer chromatographic (TLC)-densitometric procedure could effectively separate clobetasol propionate from its related compound, namely clobetasol. It is linear for clobetasol propionate in the range of 0.188 ÷ 5 µg/spot. The limit of detection (LOD) and limit of quantification (LOQ) value is 0.061 and 0.186 µg/spot, respectively. Accuracy of proposed procedure was evaluated by recovery test. The mean recovery of studied clobetasol propionate ranges from 98.7 to 101.0%. The coefficient of variation (CV, %) obtained during intra-day and inter-day studies, which was less than 2% (0.40 ÷ 1.17%), confirms the precision of described method. The assay value of clobetasol propionate is consistent with the pharmacopoeial requirements. In conclusion, it can be suitable as a simple and economic procedure for routine quality control laboratories of clobetasol propionate in topical solution.

## 1. Introduction

The corticosteroids are a group of chemical compounds which have potent immunosuppressive and anti-inflammatory activity. Topical corticosteroids are widely used for the treatment of various skin dermatoses associated with inflammation and pruritus [1,2,3,4,5,6,7]. Clobetasol 17-propionate (21-chloro-9-fluoro-11β,17-dihydroxy-16β-methylpregna-1,4-diene-3,20-dione 17-propionate) is one of the most potent topical corticosteroids [1]. It belongs to US class I of the corticosteroids, namely superpotent corticosteroids [1]. Clobetasol propionate is commercially available in different pharmaceutical formulations as ointment, cream, gel, lotion, spray, emollient cream, foam, solution for skin and shampoo in amounts about 0.05% of total weight of the formulation [3]. It is used alone or in combination with other topical agents to treat different skin dermatoses such as chronic eczema, atopic dermatoses, bullous autoimmune skin dermatoses and psoriasis [4,5,6,7]. Therefore, it is very important to establish and validate as per the International Conference on Harmonisation Guidelines (ICH) a simple and economical procedure for quantifying of clobetasol propionate in available pharmaceutical formulations [8]. The method currently used for the determination of clobetasol propionate is spectrophotometry and also RP-HPLC technique with various detection systems like for instance spectrophotometry at UV light or mass spectrometry (HPLC-MS), respectively [9,10,11,12,13,14,15,16,17,18,19,20,21]. To the best of our knowledge no much report could be found for the determination of clobetasol propionate in pharmaceutical formulation using simple and economical thin-layer chromatographic (TLC) procedure. A thorough literature review revealed that there are only few TLC methods for clobetasol propionate in respective dosage forms (i.e., cream and lotion) [22,23,24]. Until now, there has been no report regarding the use of the TLC-densitometric method for the quantification of clobetasol propionate in the form of topical solution. What is more, there is no pharmacopoeial TLC-densitometric method described for the assay of clobetasol propionate in drug formulation, including its topical solution.

Taking this consideration into account, in the present research work we propose a simple TLC-densitometric procedure for estimation and determination of clobetasol propionate in form of solution for skin (0.50 mg/mL). Established method has been carefully verified according to ICH guidelines and compared with pharmacopoeial requirements for clobetasol propionate topical solution [8,25,26]. The proposed TLC-densitometric procedure is simple to use and low-cost, thereby it can widely be applied in routine analysis and quality control of clobetasol propionate in form of topical solution.

The results obtained in this paper can confirm the suggestions set out in the current literature by the authors of other works [27,28,29], that among various analytical methods, thin-layer chromatography combined with densitometry due its simplicity and low cost is a good tool for separating, detecting and quantifying different compounds including steroids, such as selected flavonoids, polyphenols, β-carotene and food dyes in different matrices (i.e., in plant samples as well as synthetic pharmaceutical preparations) [27,28,29].

## 2. Results and Discussion

### 2.1. Procedures

#### Optimization of Chromatographic Conditions

**Figure 1:**
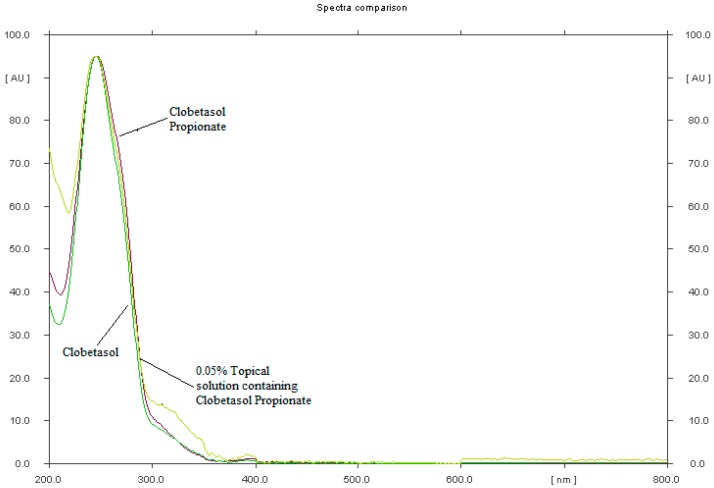
UV-VIS spectra of standard solution of clobetasol and clobetasol propionate as well as topical solution containing clobetasol propionate (λ_max_ = 246 nm).

**Figure 2:**
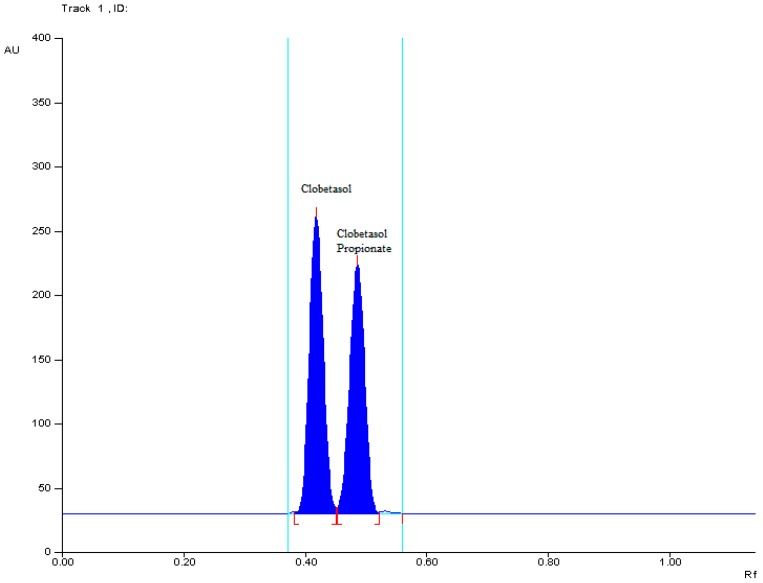
Two dimensions TLC chromatogram showing the separation of clobetasol propionate from clobetasol at *R*_F_ = 0.51 ± 0.01 and 0.45 ± 0.01, respectively.

Based on the literature review, several chromatographic systems consisted of various chromatographic plates for TLC precoated with silica gel 60F_254_ as well as silica gel RP-18F_254_ or mixture of silica gel 60 and Kieselguhr F_254_ and different mobile phases such as acetonitrile-methanol, chloroform-acetone-methanol, toluene-chloroform-methanol-ammonium and toluene-methanol in different ratio have been tried in order to find the best developing system for the analysis of clobetasol propionate in form of solution for skin [22,23,24]. Of all the chromatographic systems tested in preliminary study, the most suitable was that consisted of chromatographic plates precoated with conventional silica gel 60F_254_ as stationary phase and binary mixture of toluene-methanol (40:9.8 *v*/*v*) as a mobile phase [22]. Separation was done on preactivated at 110 °C during 20 min TLC plates. Solutions (5 μL) of clobetasol propionate and clobetasol, respectively, were spotted using Camag micropipette onto applied TLC plates. TLC chamber was saturated with mobile phase for 20 min before analysis. It was observed that saturation of TLC chamber with mobile phase for 20 min ensure good reproducibility and peak shape of examined substances. The mobile phase was run up to a distance of 7 cm, which takes about 15 min for complete development of the chromatographic plates. The plates were left to dry, then spots were scanned at maximum wavelength (λ = 246 nm), see Figure 1. An exemplary TLC densitogram of clobetasol propionate and related compound, namely clobetasol is shown in Figure 2.

The separation factors obtained on the basis of TLC densitograms using the Equations (1)–(4) for clobetasol propionate (CP) and its pharmaceutical impurity clobetasol (C) under mentioned chromatographic conditions as shown in Table 1 are satisfactory. Calculated Δ*R*_F_ for separated pair of the two examined compounds CP and C is more than 0.05. Other separation factors, such as *R*_s_, α and *R*_F_^α^ are above 1.00 (Table 1), thus indicate also good separation of CP from C. Hence, these optimum conditions were applied in subsequent work.

Δ*R*_F(1,2)_ = *R*_F2_ − *R*_F1_ (1)

where *R*_F_—retardation factor of examined compounds, and *R*_F2_ > *R*_F1_.

(2) $$R_{S}=\frac{2d}{w_{b1}+w_{b2}}$$

where *d*—distance between the centers of two neighboring bands [mm]; *w*_b_—bands width at the base line (mm).

(3) $$\alpha =\frac{\frac{1}{R_{F1}}-1}{\frac{1}{R_{F2}}-1}$$

where *R*_F1_ < *R*_F2_.

(4) $$R_{F(1,2)}^{\alpha }=\frac{R_{F1}}{R_{F2}}$$

where *R*_F1_ > *R*_F2_.

**Figure 3:**
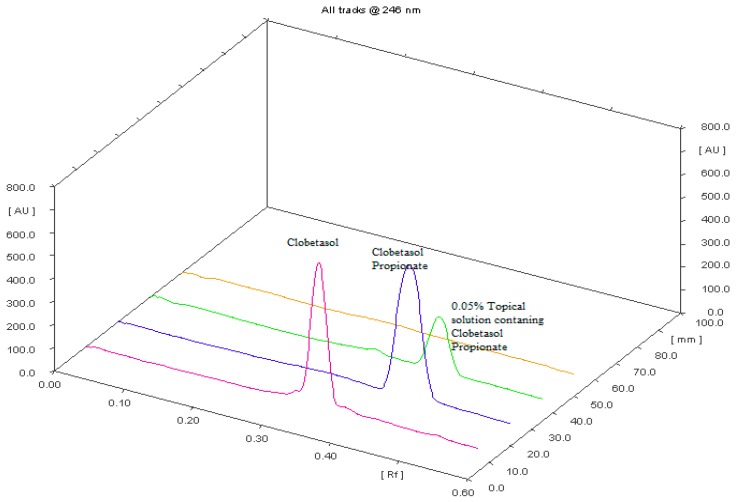
Three dimensions TLC chromatograms showing clobetasol, clobetasol propionate and marketed formulation (0.05% topical solution) of clobetasol propionate, respectively. Notes: yellow color—scanned background of TLC chromatogram; @—at.

Figure 3 performs three dimensions TLC chromatograms showing clobetasol, clobetasol propionate and marketed formulation (0.05% topical solution) of clobetasol propionate, respectively.

### 2.2. Validation Data

The results of validation studies of TLC-densitometric method adopted for the determination of clobetasol propionate in form of solution for skin using toluene-methanol (40:9.8 *v*/*v*) as the mobile phase on silica gel 60F_254_ plates with the detection at 246 nm are given below.

#### 2.2.1. Specificity

**Figure 4:**
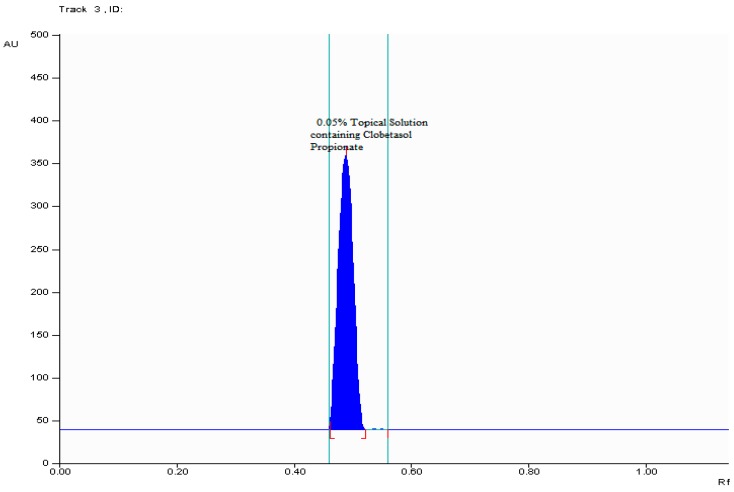
TLC chromatogram of marketed formulation (0.05% topical solution) of clobetasol propionate.

The specificity of proposed chromatographic procedure was checked by analyzing standard drug of clobetasol propionate and its marketed formulation (topical solution). The spot of clobetasol propionate in examined topical solution was confirmed by comparing *R*_F_ value and also UV-VIS spectra of both samples. Good correlation between the standard and sample peaks measured at three different regions of chromatographic bands, namely r(*S*,*M*) = 0.998 and r(*M*,*E*) = 0.994, where (*S*)—is peak start, (*M*)—peak apex and (*E*)—is peak end confirms specificity of described method. TLC densitogram of examined topical solution in Figure 4 shows that there is no excipients and additives (i.e., carbomer, isopropyl alcohol, sodium hydroxide) interference with studied active ingredient, namely clobetasol propionate present in topical solution. Therefore, the proposed procedure can be suitable in the TLC-densitometric analysis of clobetasol propionate as topical solution.

#### 2.2.2. Linearity

**Figure 5:**
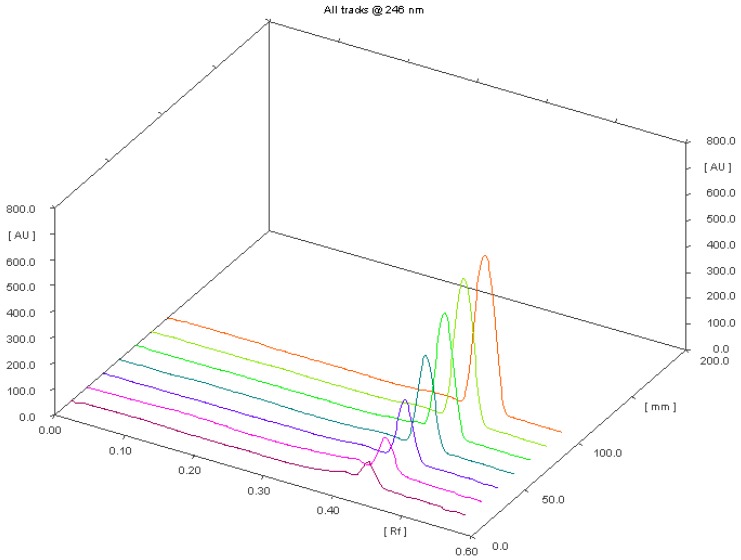
Three dimensions TLC chromatogram showing clobetasol propionate linearity range (0.188 ÷ 5 μg/spot) at *R*_F_ = 0.51 ± 0.01. Note: @—at.

**Figure 6:**
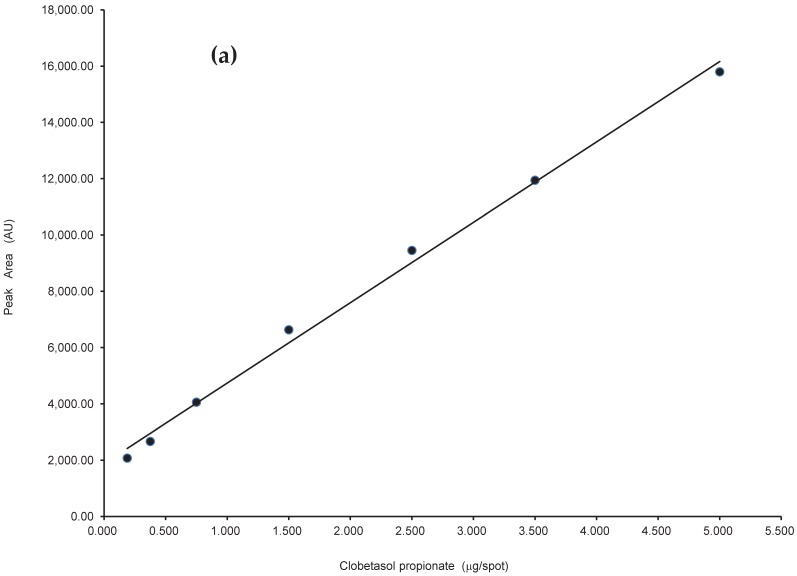

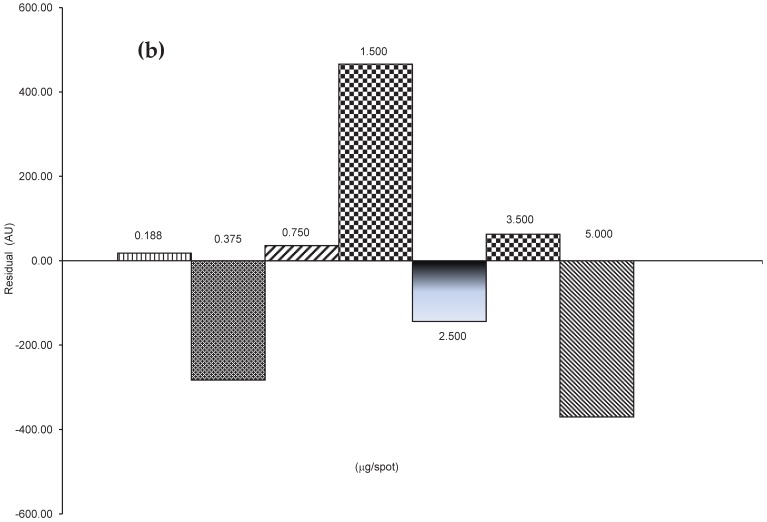
Calibration plot for clobetasol propionate (**a**) and plot of residuals (**b**) in linear working range.

Linearity of proposed method was conducted by analysis of clobetasol propionate samples at seven concentration levels: 0.188, 0.375, 0.75, 1.5, 2.5, 3.5 and 5 μg/spot—Figure 5. Each analysis was repeated three times. The calibration curves were constructed on the basis of measured peak areas against concentration of clobetasol propionate in μg/spot, Figure 6a. The linear regression equation was found to be y = 1877.43 + 2858.18x. Linearity of proposed method in a wide range of concentration of examined clobetasol propionate given in μg/spot was also assessed by defining of residuals of described linear relationship—Figure 6b. The results in Figure 6b located above as well as below *X*-axis confirm linearity of obtained calibration plot. The adopted TLC-densitometric procedure allows to quantify of clobetasol propionate in the more widely concentration range of this compound in comparison with those dedicated for other pharmaceutical formulations like for example lotion or cream [22,23].

The statistical characteristic of obtained calibration curve includes, the correlation coefficient, slope, intercept and other are summarized in Table 2. As shown Table 2 the statistical data demonstrate also strong linear relationship between variables. Correlation coefficient (r) > 0.995 fulfils the ICH requirements needed for analytical procedures used in quantifying of active ingredients [8]. Comparison with other works [9,10,11,12,13,14,15,16,17,18,19,20,21] indicates that the use of HPLC technique allows to obtain much better correlation coefficient (r ≥ 0.999) of calibration plots at lower or higher range of concentration of clobetasol propionate, respectively in relation to that described in current paper [9,10,11,12,13,14,15,16,17,18,19,20,21].

#### 2.2.3. LOD and LOQ

The limits of detection (LOD) and quantification (LOQ) of suggested method were checked and calculated on the basis of calibration plot prepared using the peak area measurements of examined clobetasol propionate at the lowest part of working range. The following Equations (5) and (6) were applied in order to calculate the LOD and LOQ value, respectively.

LOD = 3.3 × σ/*S* (5)

LOQ = 10 × σ/*S* (6)

where σ—standard deviation of the response, *S*—slope of calibration plot. The LOD was found to be 0.061 μg/spot, while the LOQ was 0.186 μg/spot. Both results confirm good sensitivity which is enough for the determination of clobetasol propionate in marketed formulation, especially in form of topical solution containing 0.50 mg of studied compound as active ingredient per one mL of analyzed topical solution. The LOD and LOQ level (given in μg/spot of clobetasol propionate), thus the sensitivity of adopted TLC-densitometric procedure is comparable to that reported for clobetasol propionate in form of cream by other authors [23]. Similar level of detection and quantitation (at micrograms) can be observed also in the case of selected HPLC procedures recommended for assay of clobetasol propionate in simple as well as complex pharmaceutical formulations, like for example in combination with salicylic acid (LOD = 0.036 μg/mL, LOQ = 0.109 μg/mL) [18]. A very low LOQ (0.75 ÷ 3 ng/mL) was achieved using the modern LC/MS/MS method for clobetasol propionate and other undeclared steroids in cosmetic creams [19].

#### 2.2.4. Accuracy

The method accuracy was determined by recovery studies and expressed as percent recovered, see Table 3. This experiment was carried out by standard addition method at different levels of clobetasol propionate in studied pharmaceutical formulation i.e., topical solution. Appropriate amount of clobetasol propionate (standard powder) corresponding to 50%, 100% and 150% of label claim had been added to the sample solution. The chromatograms were studied under the optimized chromatographic conditions. Determination was performed in triplicate at each level. Table 3 complies the results of recovery study. As is shown in Table 3, the mean value of recovery is placed in the range of 98.7% ÷ 101.0% for 50%, 100% and 150% of clobetasol propionate added. The calculated coefficient of variation (CV, %) ranges from 0.36 to 0.81, thus is less than 1%. The statistical results of recovery study are under the acceptance range according to ICH guidelines [8]. Satisfactory recovery of clobetasol propionate suggesting that there is no interference from any of excipients and additives which are present in examined topical solution. Comparison of obtained results with mean recovery of clobetasol propionate examined by proposed TLC-densitometric procedure with those obtained by HPLC system in various topical formulations indicates that the HPLC method enables achieve very similar recovery range of clobetasol propionate to the proposed TLC method. It is generally placed from 95.1 to 100.0% for propionate clobetasol in complex preparation with everolimus [15], from 97.81 to 98.35% for clobetasol propionate which has been analyzed in topical nanocapsule suspension [9] or from 100.03 to 100.05% for clobetasol propionate in its pharmaceutical preparation with salicylic acid [18].

#### 2.2.5. Precision

The precision of the proposed TLC-densitometric procedure which demonstrates the ability of an analytical method to produce consistent results was evaluated in terms of repeatability (intra-day precision) and also intermediate precision (inter-day precision) by spotting onto applied TLC chromatographic plates the sample of topical solution corresponding to 1.00, 1.50 and 2.00 μg/spot of clobetasol propionate.

Repeatability of the adopted TLC-densitometric method was performed at three different times on the same day. Inter-day variations were performed by analyzing same concentration of examined clobetasol propionate in three different days over a period of a week and by two different analysts and using two various batches of chromatographic plates. The precision of described method was expressed in terms of coefficient of variation (CV, %). The results depicted in Table 4 revealed high precision of supposed method. As shown Table 4, the mean value of recovery ranged from 98.7 to 101.5% in the case of intra-day precision. The % CV values are placed in the range between 0.49 and 0.94%, thus, less than 1%. Similarly, satisfactory results of high percent recovery of studied clobetasol propionate in topical solution ranging from 98.0 to 99.3% can be observed for measured intermediate precision (Table 4). The coefficient of variation is also good because it was found to be from 0.40 to 1.17%. Therefore, it can be suggested that the proposed TLC-densitometric method indicates relatively high intra- and also inter-day precision which is in a good agreement with ICH requirements as well as HPLC method [15,16].

Summarizing the results of accuracy and precision described above, it can be concluded that adopted method shows no significant differences in accuracy and precision when compared with reported TLC methods [22,23]. Comparability of accuracy and precision results with those obtained by selected HPLC procedures indicates that proposed TLC-densitometric method can be a good alternative for assay of clobetasol propionate in its topical solution.

#### 2.2.6. Robustness

Robustness of the proposed TLC-densitometric method was checked by evaluating the effect of small changes of applied chromatographic conditions on the results, i.e., on the measured peak area and also on the percent amount of clobetasol propionate in studied topical solution. Introduction of the following small changes into TLC procedure such as the time required for chamber saturation (±10 min), the volume of mobile phase used (±10 mL), development distance (±10 mm), the temperature of activation of chromatographic plates (±10 °C) and the time from spotting to chromatography (±10 min) was assessed. The robustness was determined at three different concentration levels of clobetasol propionate: 1.00, 1.50 and also 2.00 μg/spot. As shown Table 5, the percent assay of clobetasol propionate in examined topical solution under amended chromatographic conditions, thus for each parameter is high and ranged from 101.3% ÷ 111.8%. The % CV of peak area (found to be less than 2%). Taking into account the obtained results, it can be concluded that the adopted TLC-densitometric procedure was to be unaffected by small changes of chromatographic conditions, thereby it is robust.

#### 2.2.7. Analysis of Formulation (Topical Solution)

The accurately validated TLC-procedure in combination with densitometry was applied for determination of clobetasol propionate in commercially available topical solution containing clobetasol propionate in base at a quantity 0.50 mg/mL and others like carbomer, isopropyl alcohol, sodium hydroxide and purified water as excipients and additives, respectively. Development and quantification was performed under the mentioned chromatographic conditions. The results of assay were listed in Table 6.

The average content of six determinations was found to be 0.49 ± 0.02 mg/mL and represent 98.0% in relation to the label claim. The coefficient of variation (CV, %) is less than 2%. The results of the amount of clobetasol propionate in studied topical solution fulfil the ICH guidelines and also pharmacopoeial requirements [8,25,26]. According to USP monographs the clobetasol propionate topical solution should contain not less than 90.0% and not more than 110% of labeled amount of clobetasol propionate [26]. Good compliance of the amount of clobetasol propionate found in examined marketed formulation in form of topical solution with labeled claim as well as pharmacopoeial requirements suggesting that there is no interference from any of the excipients and additives which are normally present in clobetasol propionate topical solution, i.e., carbomer, isopropyl alcohol and sodium hydroxide. Thereby, the proposed TLC-procedure can be successfully applied for routine quality control of clobetasol propionate in form of marketed available topical solution. For comparison, the determination of clobetasol propionate in drug samples by means of HPLC method showed the results of clobetasol propionate according to the theoretical value, for example, in the range of 99.95% ÷ 102.27% or 100.71% for ointment dosage form [15,16].

## 3. Experimental Section

### 3.1. Apparatus

TLC densitometer: A Camag TLC Scanner 3 (Camag, Muttenz, Switzerland) in the reflectance absorbance mode operated by WinCATS software. The source of radiation was a deuterium and tangsten lamp respectively. The chromatographic plates were scanned with slit dimension of 8.00 mm × 0.40 mm and scanning speed of 20 mm/s. Data resolution was 100 µm/step. Spectrum scan speed was kept at 100 nm/s.

### 3.2. Chemicals and Solvents

The solvents, methanol, toluene used in current study were procured from POCh (Gliwice, Poland). Both solvents were of HPLC grade. Standard clobetasol (purity ≥ 99.5%, pharmaceutical impurity standard) was purchased from Sigma-Aldrich (St. Louis, MO, USA). Reference standard clobetasol propionate (Sigma-Aldrich) was pharmaceutical-grade substance and fulfils the requirements of USP Pharmacopoeia [26].

### 3.3. Pharmaceutical Preparation

Clobetasol propionate in form of solution for skin (bottle 50 mL) was purchased from commercial source (local market, Poland). 0.05% solution contains the active compound clobetasol propionate in quantity 0.50 mg/mL and the following excipients as well as additives: carbomer, isopropyl alcohol, sodium hydroxide and purified water.

### 3.4. Materials

Chromatography was performed on precoated silica gel 60F_254_ plates (20 cm × 20 cm, Merck, Darmstadt, Germany, Art. 1.05554), which were cut into 10 cm × 10 cm before use. The solutions were spotted onto chromatographic plates using 5 μL micropipettes (Camag, Muttenz, Switzerland). Ascending development was performed in a Camag twin-trough chamber (20 cm × 10 cm).

### 3.5. Preparation of Standard Stock Solutions

Standard stock solutions with a concentration of 1 mg/mL were prepared in methanol for clobetasol propionate and clobetasol, respectively. From the standard stock solution, different diluted solutions of clobetasol propionate were prepared. The stock solution was stored at 2–8 °C. The solution (5 μL) was spotted on the TLC plate in each case.

### 3.6. Sample Preparation

Examined topical solution (5 mL, 0.50 mg/mL) was carefully transferred into 10 mL volumetric flask, then diluted to 10 mL with methanol, and then shaken vigorously and filtered. The resulting solution at final concentration of clobetasol propionate of 0.25 mg/mL was spotted on TLC plates in quantity 5 μL. Development and quantification was performed under adopted chromatographic conditions.

## 4. Conclusions

A simple, accurate, precise, specific and robust TLC-densitometric procedure was suggested to quantify clobetasol propionate in marketed formulation in the form of a topical solution. The reported method met all ICH validation acceptance criteria. Experimental results showed that the amount of propionate clobetasol in examined pharmaceutical formulation expressed as a percentage of label claim was in good agreement with pharmacopoeial requirements for clobetasol propionate topical solution. The main advantage of supposed procedure is low cost in relation to reported HPLC method and minimum sample preparation resulting in relatively short time of analysis in comparison with TLC-densitometric and spectrophotometric procedures described for other pharmaceutical formulations such as lotion or cream, respectively. In addition to this, the validated method covers a wide range of linearity. The method showed no significant difference in accuracy and precision when compared with other reported TLC methods as well as selected HPLC procedures regarding lotion and cream analysis. The LOD and LOQ demonstrate that the sensitivity of suggested TLC-densitometric procedure is much less in relation to the modern HPLC/MS/MS systems but is enough for the purpose of routine quality control of clobetasol propionate in topical solution. The proposed TLC-densitometric procedure can be a substitute for the reported HPLC method when HPLC is not available.

## Figures and Tables

**Table 1:** Parameters of chromatographic separation of clobetasol propionate and clobetasol using proposed TLC-densitometric method.

Parameter	Results
Δ*R*_F_	0.06
*R*_S_	1.25
α	1.27
*R*_F_^α^	1.13

**Table 2:** Linear model and statistical parameters of calibration curve y = a + bx.

Parameter	Results
Wavelength (nm)	246
Linearity range (μg/spot)	0.188–5
Slope (b)	2858.18
Standard deviation of the slope (*S*_b_)	87.84
Coefficient of variation (CV, %) of slope	3.08
Intercept (a)	1877.43
Standard deviation of the intercept (*S*_a_)	226.36
Correlation coefficient (r)	0.998
Standard deviation of residuals (*S*_y/x_)	385.19
*F*	1058.85
Significance level (p)	0
Limit of detection (LOD) in μg/spot	0.061
Limit of quantification (LOQ) in μg/spot	0.186

**Table 3:** Results of accuracy.

Nominal Amount of Clobetasol Propionate in Drug (mg/mL)	Amountof Pure Clobetasol Propionate Added (mg)	Total Amount of Clobetasol Propionate in Drug Sample (mg/mL)	Average Amount of Clobetasol Propionate Found in Drug Sample (mg/mL)	±SD	CV (%)	Recovery (%)
0.50	0.25 (50%)	0.75	0.74	0.006	0.81	98.7
0.50	0.50 (100%)	1.00	1.01	0.008	0.78	101.0
0.50	1.00 (150%)	1.50	1.49	0.005	0.36	99.3

SD = standard deviation; CV = coefficient of variation.

**Table 4:** Results of method precision.

**Intra-Day Precision (Repeatability, *n* = 3)**			
**Amount of Clobetasol Propionate (µg/spot)**	**Measured Amount ± SD**	**CV (%)**	**Recovery (%)**
1.00	1.01 ± 0.008	0.79	101.0
1.50	1.48 ± 0.014	0.94	98.7
2.00	2.03 ± 0.010	0.49	101.5
**Intra-Day Precision (Intermediate Precision, *n* = 3)**			
**Amount of Clobetasol Propionate (µg/spot)**	**Measured Amount ± SD**	**CV (%)**	**Recovery (%)**
1.00	0.98 ± 0.009	0.92	98.0
1.50	1.49 ± 0.006	0.40	99.3
2.00	1.97 ± 0.023	1.17	98.5

SD = standard deviation; CV = coefficient of variation.

**Table 5:** Robustness of proposed TLC-densitometric method (*n* = 3).

Method Parameters	CV (%) of Peak Area	Assay of Clobetasol Propionate (%)
Chamber saturation time (±10 min)	0.75	101.5
Volume of mobile phase (varied ±10 mL)	1.48	111.8
Development distance (±10 mm)	1.31	109.1
Temperature of activation of chromatographic plates (±10 °C)	0.87	101.3
Time from spotting to chromatography (±10 min)	0.97	104.8

CV = coefficient of variation.

**Table 6:** Assay of clobetasol propionate by proposed TLC-densitometric method in topical solution.

Parameter	Data
Number of determinations	6
The label claim of clobetasol propionate in (mg/mL)	0.50
Average amount of clobetasol propionate (mg/mL)	0.49
Minimum amount of clobetasol propionate (mg/mL)	0.47
Maximum amount of clobetasol propionate (mg/mL)	0.51
Standard deviation (SD)	0.008
Coefficient of variation (CV, %)	1.63
Confidence interval of arithmetic mean with confidence level equal 95% (mg/mL)	0.49 ± 0.02
Amount of clobetasol propionate (%) in relation to the label claim	98.0%
